# Elective Laparoscopic Cholecystectomy Performed by Residents: Impact on Operative Time and Surgical Outcomes

**DOI:** 10.7759/cureus.114709

**Published:** 2026-08-18

**Authors:** Ana Cabral, Andreia Silva, Bruno Couto, Maria Assuncao, Catarina Rodrigues

**Affiliations:** 1 General Surgery, Hospital da Horta, Entidade Pública Empresarial Regional (EPER), Horta, PRT

**Keywords:** cholecistectomy, general surgery, internship and residency, laparoscopic cholecystectomy, patient safety

## Abstract

Background: Operative autonomy is fundamental in general surgery training. Concerns have been raised regarding the adequacy of operative exposure during general surgery residency, with many graduating residents perceived as insufficiently prepared. Reduced opportunities for progressive operative autonomy have been identified as a contributing factor. This study aimed to compare the outcomes of elective laparoscopic cholecystectomies (LCs) performed by residents and by attending surgeons in a Portuguese institution.

Methods: A retrospective review of all elective LCs performed between January 2020 and December 2023 was conducted. Demographic, clinical and perioperative variables were collected.

Results: A total of 189 elective LCs were included, of which 46.6% were performed by residents in a supervised manner. Baseline demographic and clinical characteristics were comparable between groups. Resident-performed procedures had longer operative times (101 vs. 70 minutes, p = 0.003), while postoperative length of stay was shorter (0 vs. 1 day, p = 0.020). No significant differences were observed in intraoperative findings, conversion to open surgery, major postoperative complications, 30-day readmission or late postoperative outcomes. In multivariable analysis, resident participation remained independently associated with longer operative time (β = 13.46 minutes, p = 0.028), together with BMI >25 kg/m² and gallbladder adhesions.

Discussion: Supervised resident participation in elective LCs was independently associated with a modest increase in operative time without increasing perioperative morbidity. These findings support resident autonomy under appropriate supervision and adherence to the national general surgery residency programme, suggesting that the additional operative time is an acceptable trade-off for surgical training while maintaining patient safety.

## Introduction

Progressive operative autonomy is a fundamental component of general surgery training, allowing residents to acquire the technical skills, decision-making ability and confidence required for independent practice [1].

Despite its importance, concerns have emerged regarding the adequacy of operative exposure during general surgery residency. Surveys of fellowship directors and practicing surgeons have suggested that many graduating residents are insufficiently prepared for independent practice, particularly regarding autonomy and technical skills [2,3].

This perceived decline in operative preparedness has been attributed to multiple factors, including work-hour balance, increasing administrative responsibilities, growing procedural complexity and reduced surgical opportunities [2-6]. A Portuguese nationwide survey showed that residents' perceived preparedness for independent practice was associated with operative experience rather than emergency department exposure or study time [7].

One of the challenges in surgical training is ensuring that residents acquire autonomy in increasingly complex procedures without compromising patient safety. One example is laparoscopic cholecystectomy (LC), which is a routinely performed procedure but requires good surgical technique and accurate identification of the biliary anatomy. Bile duct injury remains an uncommon but potentially devastating complication [8], emphasizing the need for structured training, appropriate supervision and strict adherence to safe dissection principles. A more recent nationwide survey of Portuguese general surgery residents highlighted concerns regarding biliary surgery training, with all participants identifying the need for additional training in complex biliary procedures, despite widespread exposure to LC [9]. Although the Portuguese general surgery residency is a national six-year standardized programme, differences in operative exposure and supervised surgical autonomy may exist between institutions, reflecting variations in case volume and local training practices.

The present study aimed to compare outcomes of elective LCs performed by residents and by attending surgeons in a Portuguese peripheral hospital.

## Materials and methods

Study setting

A retrospective review was conducted of all elective LC procedures performed between January 2020 and December 2023 at our institution. This institution is a peripheral secondary-level hospital, serving a population of approximately 50,000 inhabitants, with an estimated level of 50 elective LCs annually, most of them admitted as day-case procedures. The department’s residents include first-, second-, and sixth-year (senior) residents. The intermediate years of training are completed at higher-volume referral centres, reflecting the institution's partial accreditation for General Surgery residency training by the Portuguese Board of General Surgery.

The department's usual practice is that, during the first year of residency, trainees acquire fundamental laparoscopic skills through less technically demanding procedures, such as laparoscopic appendectomies. LC training usually begins in the second year of residency, initially with selected operative steps, including dissection of the gallbladder from the liver bed, followed by progressive dissection of Calot's triangle and achievement of the Critical View of Safety. As residents demonstrate increasing technical proficiency and sound intraoperative judgment, they progressively perform the entire procedure under decreasing levels of supervision. Accordingly, in the present study, procedures were classified as resident-performed only when the resident acted as the primary surgeon and completed the operation under passive assistance or supervision alone by the attending surgeon (broadly corresponding to the highest levels of the Zwisch Scale).

The study included all consecutive adult patients (18 years old or older) who underwent elective LCs performed in the above-mentioned time period. Cases requiring conversion were also included in the analysis. Exclusion criteria included pregnancy, LC performed during a surgery for another indication and previous biliary reconstruction.

Variables

We collected the following variables: demographic (age, sex, body mass index (BMI) and American Society of Anesthesiologists (ASA) physical status classification); preoperative (surgical indication, previous abdominal surgery, preoperative imaging findings, time to surgery (since enrollment in the waiting list), previous endoscopic retrograde cholangiopancreatography (ERCP)), intraoperative (operating surgeon (resident or attending), surgical setting (day-case or inpatient), operative time, gallbladder adhesions and wall thickening, gallbladder rupture, bile duct injury, documentation of the Critical View of Safety and bailout procedure) and postoperative variables (length of hospital stay, conversion to inpatient admission, readmission within 30 days, persistent postoperative symptoms, postoperative ERCP, early postoperative complications (≤30 days, according to the Clavien-Dindo classification [10]) and late postoperative complications (>30 postoperative days)).

Data were obtained through a review of electronic medical records. Our institution is the sole hospital in its referral area, making it highly likely that patients experiencing early or late postoperative complications would eventually be admitted at our center, thereby minimizing loss of clinically relevant follow-up (except in the uncommon event of relocation).

All patients had a minimum follow-up of 18 months from the date of surgery.

Statistical analysis

Categorical variables are presented as frequencies and percentages. Continuous variables were assessed for normality using the Shapiro-Wilk test and visual interpretation of the histograms. Variables are presented as mean and standard deviation (SD) or median and lower (Q1) and upper (Q3) quartiles, as appropriate. Statistical analysis was performed using the Chi-square test, Fisher's exact test, Student's t-test and the Mann-Whitney U test, according to the variable type and distribution of the data. A multivariable linear regression model was made to identify independent predictors of prolonged operative duration. A two-sided p value of <0.05 was considered statistically significant. All analyses were performed using IBM® SPSS® Statistics, version 30.0 (IBM Corp., Armonk, NY, USA). 

Ethics

The study was approved by the Institutional Ethics Committee (approval number 06/2026) and conducted in accordance with the Declaration of Helsinki.

## Results

A total of 189 patients undergoing elective LC were included in the study. Symptomatic cholelithiasis was the indication for surgery in the vast majority of patients, with three patients undergoing cholecystectomy for a gallbladder polyp and one for gallbladder adenomyomatosis. Of the 189 cases, 88 (46.6%) were performed by second- or sixth-year surgical residents in a supervised manner. Baseline demographic and clinical characteristics of the patients are summarized in Table 1. 

**Table 1:** Patient characteristics according to primary surgeon (N = 189). Total number of participants = 189. Statistical significance was defined as p <0.05. *Percentages calculated excluding missing values. ^‡^Mann-Whitney U test. ^§^Chi-squared test.^ †^Fisher's exact test. U = Mann-Whitney U statistic. χ² = Chi-square statistic. ASA = American Society of Anesthesiologists. ERCP = endoscopic retrograde cholangiopancreatography.

	Resident (n = 88)	Surgeon (n = 101)	p-value	U	χ²
Female gender, n (%)	70 (79.5)	71 (70.3)	0.143^§​​​​​​^		2.123
Age (years), median (Q1, Q3)	52.5 (41, 68)	55 (43, 67)	0.811^‡^	4354.5	
BMI > 25 kg/m², n (%)*	56 (68.3)	53 (59.6)	0.235^§​​​​​​^		1.411
Dyslipidaemia, n (%)	28 (31.8)	40 (39.6)	0.266^§​​​​​​^		1.238
Diabetes mellitus, n (%)	15 (17.0)	16 (15.8)	0.824^§​​​​​​^		0.050
Hypertension, n (%)	43 (48.9)	44 (43.6)	0.466^§​​​​​​^		0.532
Previous abdominal surgery*	22 (25.9)	33 (33.0)	0.291^§​​​​​​^		1.114
ASA class III, n (%)	19 (22.6)	23 (23.5)	0.892^§​​​​​​^		0.018
Preoperative ERCP, n (%)	2 (2.3)	6 (5.9)	0.288^†^		
Time to surgery (days), median (Q1, Q3)	123 (55, 185)	103 (56, 179)	0.710^‡^	4261.5	
Outpatient admission, n (%)	50 (56.8)	51 (50.5)	0.385^§​​​​​​^		0.756

Patients in the two groups were comparable regarding age, sex, body mass index, comorbidities, previous abdominal surgery, ASA physical status, preoperative ERCP, time to surgery and outpatient admission status, with no statistically significant differences between groups (all p > 0.05).

Intraoperative outcomes

Operative findings and intraoperative outcomes are summarized in Table 2. Procedures performed by residents were associated with significantly longer operative times than those performed by attending surgeons (median 101 (69-122) vs. 70 (50-114) minutes, p = 0.003). No significant differences were observed regarding the presence of gallbladder adhesions or gallbladder wall thickening, the occurrence of iatrogenic gallbladder perforation, bile duct injury, achievement of the critical view of safety or the need for bailout procedures.

**Table 2:** Operative findings and intraoperative outcomes according to primary surgeon (N = 189). Total number of participants = 189. Operative time was available for 187 patients (88 residents and 99 surgeons). Statistical significance was defined as p <0.05. ^‡^Mann-Whitney U test. ^§^Chi-squared test. ^†^Fisher's exact test. U = Mann-Whitney U statistic. χ² = Chi-square statistic.

	Resident (n = 88)	Surgeon (n = 101)	p-value	U	X2
Operative time (minutes), median (Q1, Q3)	101 (69, 122)	70 (50, 114)	0.003 ^‡^	3253.0	
Gallbladder adhesions, n (%)	21 (23.9)	35 (34.7)	0.105 ^§^		2.626
Gallbladder wall thickening, n (%)	3 (3.4)	9 (8.9)	0.122 ^§^		2.394
Gallbladder perforation, n (%)	20 (22.7)	30 (29.7)	0.278 ^§^		1.176
Bile duct injury, n (%)	0 (0.0)	1 (1.0)	1.000^†^		
Critical view of safety, n (%)	88 (100.0)	96 (95.0)	0.062^†^		
Bailout procedure, n (%)	0 (0.0)	4 (4.0)	0.125^†^		

Postoperative outcomes

Postoperative outcomes are summarized in Table 3. Patients operated on by attending surgeons had a longer postoperative hospital stay than those operated on by supervised residents (median 1 (0-2) vs. 0 (0-1) days, p = 0.020). There were no significant differences between groups regarding conversion from outpatient to inpatient admission, major postoperative complications during the first 30 postoperative days (Clavien-Dindo grade ≥III), 30-day readmission, persistent postoperative symptoms or late postoperative complications. Postoperative ERCP was performed more frequently in patients operated on by attending surgeons (6.9% vs. 0.0%, p = 0.015); however, all postoperative ERCPs were performed for residual common bile duct stones. Late postoperative complications were mainly small incisional hernias, but included one case of death in the intensive care unit, in the same hospital stay, due to septic shock. In this sample, all bailout procedures happened to be conversion to open surgery.

**Table 3:** Postoperative outcomes according to primary surgeon (N = 189). Statistical significance was defined as p <0.05. ^ ‡^Mann-Whitney U test. ^†^Fisher's exact test. U = Mann-Whitney U statistic. ERCP = endoscopic retrograde cholangiopancreatography.

	Resident (n = 88)	Surgeon (n = 101)	p-value	U
Length of stay (days), median (Q1, Q3)	0 (0, 1)	1 (0, 2)	0.020^‡^	3627.0
Conversion to inpatient admission, n (%)	3 (3.4)	8 (7.9)	0.225^†^	
Clavien-Dindo ≥III, n (%)	1 (1.1)	5 (5.0)	0.218^†^	
Readmission within 30 days, n (%)	0 (0.0)	4 (4.0)	0.125^†^	
Persistent postoperative symptoms, n (%)	2 (2.4)	5 (5.2)	0.450^†^	
Postoperative ERCP, n (%)	0 (0.0)	7 (6.9)	0.015^†^	
Postoperative complications (>30 days), n (%)	5 (5.7)	4 (4.0)	0.735^†^	

Multivariable analysis of operative time

Given the significant difference in operative time between groups, a multivariable linear regression analysis was performed to identify factors independently associated with operative duration (Table 4). The model was performed adjusting for age, BMI >25 kg/m², ASA class III, gallbladder adhesions, gallbladder wall thickening and surgery performed by residents. Residents remained independently associated with a longer operative time (β = 13.46 minutes, 95% CI 1.46-25.46; p = 0.028). BMI >25 kg/m² (β = 18.82 minutes, 95% CI 6.23-31.42; p = 0.004) and the presence of gallbladder adhesions (β = 28.93 minutes, 95% CI 15.03-42.84; p < 0.001) were also independently associated with prolonged operative duration. Furthermore, gallbladder wall thickening showed a borderline association with prolonged operative time (p = 0.051).

**Table 4:** Multivariable linear regression analysis of factors associated with operative time. Model statistics: R² = 0.250; adjusted R² = 0.221; F = 8.62; p < 0.001. Adjusted β coefficients represent the estimated difference in operative time (minutes). The model included 162 complete cases. Statistical significance was defined as p <0.05. ASA = American Society of Anesthesiologists.

	β coeficient (min)	95% CI	p-value
Resident performed surgery	13.46	1.46-25.46	0.028
Age (per year)	0.13	-0.30-0.57	0.544
BMI > 25	18.82	6.23-31.42	0.004
ASA class III	12.80	-2.69-28.29	0.105
Gallbladder adhesions	28.93	15.03-42.84	<0.001
Gallbladder wall thickening	24.87	-0.10-49.85	0.051

## Discussion

The present study demonstrates that elective LCs performed by residents, supervised by attending surgeons, is associated with a modest increase in operative time while achieving comparable intraoperative and postoperative outcomes. After adjustment for potential confounders, resident participation remained independently associated with an approximately 13-minute increase in operative duration. Importantly, no increase in major complications, bile duct injury, conversion to open surgery or readmission was observed.

Our findings are consistent with those reported by Sousa et al. [11], who evaluated more than 1,000 elective LCs. They found that residents as main surgeons were associated with a modest increase in operative time (approximately three minutes for second-year residents and 10 minutes for first-year residents), without significant differences in intraoperative or postoperative outcomes or adverse events. Similarly, in a series of 569 elective LCs reported by Pariani et al. [12], residents were associated with an approximately 18-minute increase in operative time, without significant differences in intraoperative or postoperative outcomes. The authors also demonstrated shorter operative times among senior compared with junior residents, highlighting the expected learning curve during surgical training.

In our institution, attainment of the Critical View of Safety according to Strasberg's principles is a mandatory step during LC and is routinely documented in the operative report. While current evidence increasingly supports image documentation of the Critical View of Safety whenever feasible [13], photographic or video recording of the Critical View of Safety is not performed as part of our standard practice; its achievement is confirmed intraoperatively by the attending surgeons together with the residents before ligation of the cystic duct and artery.

Major complications (Clavien-Dindo ≥III) were infrequent in both groups. Although the absolute number was lower among resident-performed procedures, the difference was not statistically significant. 

The only Clavien-Dindo grade IVb complication occurred in a patient with multiple major comorbidities, including liver cirrhosis, morbid obesity, type 2 diabetes mellitus, chronic kidney disease, obstructive sleep apnea and hyperuricemia, who also underwent a technically demanding procedure. This procedure was performed by an attending surgeon rather than a resident, required conversion to open surgery, and despite postoperative intensive care management the patient died during the same hospital admission.

Multivariable analysis demonstrated that resident participation remained independently associated with a modest increase in operative time of approximately 13 minutes, even after adjustment for patient- and procedure-related factors. Obesity and the presence of gallbladder adhesions were also independent predictors of prolonged surgery, reflecting greater operative complexity. Importantly, although residents as main surgeons increased operative duration, this did not translate into higher perioperative morbidity, suggesting that the additional operative time represents an acceptable trade-off for supervised surgical training.

Several factors may explain these findings. In our institution, resident autonomy is introduced progressively according to both the year of training and the individual resident's technical competence and experience, with all procedures performed under the direct supervision of attending surgeons. Furthermore, LC is performed according to standardized operative principles, particularly routine confirmation of the Critical View of Safety before division of the cystic structures, which likely contributed to the low complication rates observed in both groups.

Taken together, our findings indicate that supervised resident participation in elective LC is associated with a modest increase in operative time without adversely affecting the safety of the procedure and other postoperative outcomes. These results reinforce existing evidence supporting progressive resident autonomy under appropriate supervision and suggest that the additional operative time is an acceptable trade-off for the acquisition of surgical competence.

Strengths and limitations

This study has several limitations. Its retrospective single-center design may have introduced selection and information bias, and the sample size may have limited the statistical power to detect differences in infrequent outcomes. The patient characteristics and the intraoperative findings, including gallbladder adhesions, wall thickening and Critical View of Safety achievement, were extracted from medical records and operative reports; therefore, they depended on the accuracy of surgical documentation. Furthermore, because residents performed procedures under direct supervision, more challenging operations may have been taken over by attending surgeons according to intraoperative judgment. This potential crossover could have introduced selection bias and contributed to longer operative times in the attending surgeon group, thereby underestimating the independent effect of residents as main surgeons. Finally, as in all observational studies, residual confounding cannot be completely excluded despite adjustment for relevant clinical variables in the multivariable analysis.

Despite these limitations, this study has several strengths. It included a consecutive cohort of patients undergoing elective LC within a single institution, ensuring a standardized surgical and training environment, and a direct comparison between resident- and attending-performed procedures. In addition to operative time, a comprehensive range of clinically relevant intraoperative and postoperative outcomes was evaluated, and multivariable regression analysis was performed, strengthening the validity of the observed association between resident participation and operative duration. Finally, the study reflects real-world surgical training, where residents operate under direct consultant supervision, enhancing the clinical applicability of the findings.

## Conclusions

In conclusion, our findings suggest that supervised resident participation in elective LC is safe and should not be discouraged on the basis of concerns regarding patient outcomes. The modest increase in operative time observed in resident-performed cholecystectomies appears to be a reasonable trade-off for the development of surgical competence, particularly given that no increase in perioperative morbidity was observed, supporting the continued implementation of progressive resident autonomy under appropriate supervision.
